# Organoid systems to study the human female reproductive tract and pregnancy

**DOI:** 10.1038/s41418-020-0565-5

**Published:** 2020-06-03

**Authors:** Lama Alzamil, Konstantina Nikolakopoulou, Margherita Y. Turco

**Affiliations:** 1 Department of Pathology, University of Cambridge, Tennis Court Road, Cambridge, CB2 1QP UK; 2 Centre for Trophoblast Research, Downing Street, Cambridge, CB2 3EG UK

**Keywords:** Cell biology, Physiology, Diseases

## Abstract

Both the proper functioning of the female reproductive tract (FRT) and normal placental development are essential for women’s health, wellbeing, and pregnancy outcome. The study of the FRT in humans has been challenging due to limitations in the in vitro and in vivo tools available. Recent developments in 3D organoid technology that model the different regions of the FRT include organoids of the ovaries, fallopian tubes, endometrium and cervix, as well as placental trophoblast. These models are opening up new avenues to investigate the normal biology and pathology of the FRT. In this review, we discuss the advances, potential, and limitations of organoid cultures of the human FRT.

## Abstract (web summary)

■.

## Graphical abstract

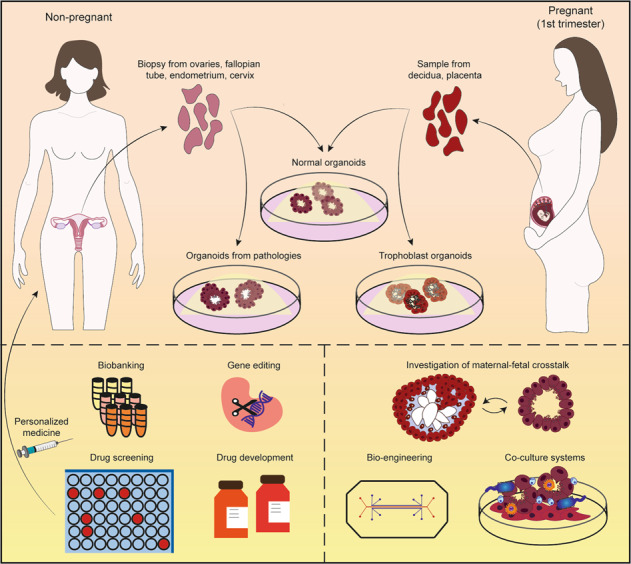

## Facts

- The efficient and coordinated function of the FRT is essential for reproduction and women’s wellbeing. Perturbations in these processes are the cause of a range of disorders from infertility to cancer.
- Organoids can be derived from healthy and pathological tissues of the FRT.
- Organoids of the reproductive tract faithfully recapitulate key morphological, functional, and molecular characteristics of their cells of origin.
- The organoid systems provide an essential tool to study the physiology and disease of the FRT.

## Open questions

- How well do FRT organoids model the cellular heterogeneity of the tissue of origin?
- Are the different cell states across the menstrual cycle represented in the FRT organoid models?
- What are the signaling pathways and transcriptional networks regulating proliferation and differentiation of the organoids of the FRT?
- Can FRT organoids be used to generate more complex models that incorporate stromal and immune cell types?
- Can early developmental processes of pregnancy be modeled by co-culture of FRT organoids with embryos or trophoblast organoids?

## Introduction

The female reproductive tract (FRT) develops during fetal and early postnatal life [1]. It is derived principally from the Müllerian ducts (also referred to as the paramesonephric ducts) that develop from the intermediate mesoderm of the urogenital ridge on either side of the midline during 6–9.5 gestational weeks in humans [2]. Initially, this structure is present in both sexes, but the secretion of anti-Müllerian hormone results in regression of the Müllerian ducts in males [3]. In contrast, in females, they differentiate to develop into the fallopian tubes, uterus, cervix, and upper vagina. The ovaries are derived from the genital ridge which forms on the medial side of the urogenital ridge. The proper development and functioning of the FRT are essential for it to fulfill its ultimate goal of reproduction that includes the physiological processes of oocyte maturation, fertilization, implantation, fetal growth, and parturition.

Disorders of FRT that include carcinomas of the cervix and endometrium, endometriosis, infertility, and heavy menstrual bleeding are alarmingly common and are a source of major suffering [4]. Effective treatments are still lacking for many of these conditions. Furthermore, factors such as obesity and delaying the age of reproduction have led to an increase in prevalence in infertility and uterine cancers [5, 6]. Thus, understanding the biology of the FRT is becoming of increasing importance.

Neither the biology of the normal human FRT nor the etiology of most of its disorders is clearly understood because they have been difficult to study ex vivo*.* The most commonly used in vitro models include primary cells, cancer cell lines, tissue explants, and organotypic cultures [7–59] (Table 1). However, they have limitations. Primary cells from tissue biopsies cannot be propagated indefinitely and lose their epithelial phenotype. Available cell lines are often derived from cancers or are immortalized and do not represent cells in their normal physiological state. Although cell lines derived from endometrial (e.g., Ishikawa, ECC-1) [26, 60], cervical (e.g. HeLa [30], SiHa [31], C33a [32]), and ovarian carcinomas [7, 61, 62] have been essential tools to study these tumors, they do not maintain the original cellular heterogeneity due to selection of cells with proliferative advantage. Furthermore, culturing cells in two-dimensional (2D) monolayers deprives them of the three-dimensional (3D) environmental stimuli from the surrounding matrix that play a major role in cellular behavior [63]. The study of the physiology of pregnancy in humans has also been a major challenge because, compared with other organs, the reproductive tract and its association with the placenta are the most evolutionary diverse across species. Many of the human-specific features are modeled only to a limited extent in animal models and available in vitro tools. Thus, long-term physiologically relevant models that recapitulate epithelial architecture, cellular heterogeneity and functionality of the different regions of the human FRT are essential.

**Table 1:** In vitro models of the human FRT used before the establishment of organoid systems.

Cell culture system	Tissue	Model	Reference^a^
2D	Ovaries	Tissue-derived ovarian surface epithelial cultures	[7–9]
		Immortalized ovarian surface epithelial cultures	[10–12]
	Fallopian tubes	Tissue-derived epithelial cultures	[13]
		Immortalized epithelial cultures	[14–16]
	Endometrium	Tissue-derived epithelial cultures	[17–19]
		Tissue-derived stromal cultures	[18, 20]
		Tissue-derived endothelial cultures	[21–23]
		iPSC-derived stromal cultures	[24]
		Carcinoma-derived cell lines (HEC-1, Ishikawa, RL95–2, St-1b, ECC-1)	[25–29]
	Cervix	Tissue-derived epithelial cultures	[30–32, 34, 35]
		HPV16-immortalized epithelial cell line	[36]
		Carcinoma-derived cell lines (HeLa,SiHa,C33a,CaSki,ME-180)	[33, 37]
3D	Ovaries	Tissue-derived epithelial spheroid cultures	[38, 39]
	Fallopian tubes	Tissue-derived epithelial spheroid cultures	[40]
		iPSC-derived epithelial cell 3D cultures	[41]
	Endometrium	Tissue-derived epithelial cell 3D cultures	[42]
		Organotypic cultures from endometrial tissue	[43–46]
		Mesenchymal-derived epithelial-like cell 3D cultures	[47]
		Spheroids derived from endometrial adenocarcinoma cell lines	[48]
		Spheroids of mesenchymal stem cells derived from menstrual blood/endometrial fragments	[49]
	Cervix	Organotypic cultures from primary cervical epithelial cells	[50, 51]
		Organotypic cultures from HPV16-immortalized cells	[50]
Tissue explants	Ovaries	Ovarian tissue explants	[52]
	Fallopian tubes	Fimbria explants	[53, 54]
	Endometrium	Non-pregnant endometrium explants	[55, 56]
		1st trimester decidua parietalis explants	[57, 58]
	Cervix	Cervical explants	[59]

A summary of the different in vitro tools available for studying the biology of human FRT (excluding the recently derived organoid models). The models have been grouped according to the culture type: 2D monolayer cultures, 3D models and tissue explants. A few representative references are cited per type of model.

^a^Reprentative references of each model.

 Culturing cells in 3D is a technique that has been employed for decades using a variety of methods, but these were not chemically defined nor standardized (Table 1). In 2009, Sato and Clevers described a 3D culture system that has led to the systematic generation of organoids from many different organs [64]. Organoids are 3D cellular structures that retain functional and morphological features of tissues [65]. They can be derived from tissues or pluripotent stem cells. For the generation of organoids from tissues, primary cells are embedded into a hydrogel containing extracellular proteins, usually the commercially available Matrigel isolated from Engelbreth-Holm-Swarm (EHS) mouse sarcoma, which acts as its basement membrane. Cells are cultured in a medium that recapitulates signals from the specific niche for each tissue. The organoid technology has several major advantages: long-term propagation of primary cells; recapitulation of the molecular, and functional characteristics of the tissue; genetic stability over time; freezing/thawing allowing bio-banking; and the ability to manipulate experimentally with a range of approaches [65]. Organoid technology is now transforming the way we study the FRT in physiology and disease.

**Fig. 1:**
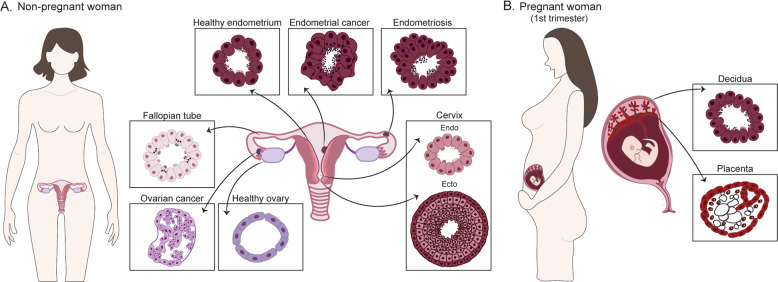
Tissue-derived organoids of the human FRT and placenta. **a** Types of organoids derived from the human FRT using tissue samples (normal and pathological) from non-pregnant women: ovaries, fallopian tubes, endometrium, and cervix. Organoids can also be derived from disorders of the FRT such as endometriosis and cancer. The different organoid systems show specific features that recapitulate the epithelial organization of their tissue of origin. **b** Organoid systems that have been derived from the pregnant endometrial lining (decidua) and the first-trimester placenta (fetal origin).

Here, we provide a general overview of the female reproductive system and describe the organoid models available to study its biology in health (including pregnancy) and disease (Fig. 1). We focus on organoids derived from human adult tissues. We include trophoblast organoids as, although the placenta is an organ of fetal origin and is not part of the FRT, it is closely associated as a functional unit with the uterine lining, the decidua, during pregnancy.

## Organoids for the study of tissue physiology of the FRT and placenta

### Ovaries

**Fig. 2:**
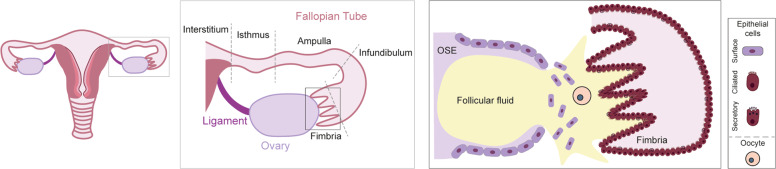
Anatomical relationship of the ovary and the fallopian tube at the time of ovulation. The fallopian tubes consist of: the fimbriae, projections bordering the ovaries; the infundibulum, opening to which fimbriae are attached; the ampulla, the longer section of the tube where fertilization usually occurs; the isthmus, narrow section adjacent to the uterine cavity and the interstitium that extends from the uterine cavity through the uterine muscle (central box). The ovaries are encapsulated by the single-cell layered ovarian surface epithelium (OSE). The fimbria of the fallopian tube envelops the ovaries and consists of the columnar ciliated and secretory epithelium. During ovulation, the OSE ruptures resulting in the release of follicular fluid and the oocyte into the fimbria. The oocyte will travel down the fallopian tube guided by the movement of the cilia where it will be fertilized by a sperm cell (right box).

The ovaries are responsible for the cyclical maturation and release of oocytes into the fallopian tubes. They also function as endocrine glands, secreting estrogen and progesterone, that act together to coordinate the cyclical changes in the endometrium [66]. In mammals, the ovaries are attached to the uterus by the ovarian ligaments. Like other organs of the reproductive tract, the ovaries are dynamic as demonstrated by the cyclic rupture and repair of the ovarian surface epithelium (OSE) [67]. Under the influence of pituitary gonadotropins (follicle-stimulating hormone and luteinizing hormone), the ovarian follicles grow and ultimately the oocyte is released from the ruptured OSE at ovulation. The released oocyte is collected by ciliated cells in the fimbria of the fallopian tubes (Fig. 2). Post ovulation, the OSE proliferates in order to efficiently repair the wound [67].

No long-term cultures of the healthy ovarian epithelium have yet been reported. The first 3D cultures of OSE were established to investigate the link between chronic inflammation and ovarian cancer [68]. Tissue-derived cultures of OSE from women with benign gynecological diseases were seeded on Matrigel-coated wells overlayed with 2% Matrigel in a medium based on 3D cultures of mammary epithelial acini. Single cell-layered epithelial spheroids formed that were surrounded by a basement membrane, containing a central lumen. The spheroids showed apical polarity but failed to grow long-term. Continuous treatment of the spheroids with TNFα induced a precancerous phenotype with increased proliferation and loss of apicobasal polarity. More recently, 3D cultures from OSE have been derived from carriers at high risk of ovarian cancer due to *BRCA1/BRCA2* mutations, who had undergone prophylactic bilateral salpingo-oophorectomy (Tables 2 and 3). The organoids are of epithelial origin (KRT8^+^) and have a cystic morphology with epithelial invaginations. Although they express the proliferative marker Ki67, their growth is slow with cultures becoming confluent after 2–3 weeks [69].

**Table 2:** Methods for the derivation of organoids from healthy tissues of the human FRT and the placenta.

Type	Tissue source	Digestion method	Substrate	Reference
Ovarian organoids	Biopsies from women with/without *BRCA1*/*BRCA2* mutations undergoing prophylactic bilateral salpingo-oophorectomy	Mechanical (mincing) and enzymatic (Collagenase XI with ROCK inhibitor Y-27632)	10 mg/ml Cultrex® Basement Membrane Matrix, Type 2	[69]
Fallopian tube organoids	Anatomically normal biopsies from women with benign gynecological disease	Enzymatic (Collagenase I) and mechanical (scraping)	100% Matrigel®	[71]
Endometrial organoids	Decidua in 8–12 weeks gestation from normal pregnancy	Mechanical (mincing) and enymatic (Dispase II and Collagenase V)	100% Matrigel®	[78]
	Endometrium in secretory, proliferative, and postmenopausal phase from non-hormonally treated women	Mechanical (mincing) and enzymatic (Dispase II and Collagenase V)	100% Matrigel®	[78]
	Endometrium in proliferative phase from non-hormonally treated women	Mechanical (mincing) and enzymatic (Collagenase IV and TrypLE with Y-27632)	70% Matrigel®	[77]
	Decidua in 8–11 weeks gestation from normal pregnancy	Mechanical (mincing) and enzymatic (Collagenase I with DNase I)	60% Matrigel®	[79]
Cervical organoids	Anatomically normal biopsies	Mechanical (mincing) and enzymatic (Collagenase II and TrypLE Express)	100% Matrigel®	[94]
Trophoblast organoids	Placenta in 6–7 weeks gestation from normal pregnancy	Mechanical (chopping) and enzymatic (Trypsin with DNase I)	60% Matrigel®	[100]
	Placenta in 6–9 weeks gestation from normal pregnancy	Mechanical (scraping) and enzymatic (Trypsin-EDTA and collagenase V)	100% Matrigel®	[101]

The type of tissue, methods used for isolating, and culturing primary cells for the establishment of organoids from normal human FRT tissues are listed.

**Table 3:** Medium components for the culture of organoids derived from the human FRT and placenta.

Type	Expansion medium components^a^	Differentiation medium components	Reference
Ovarian	**W**nt CM, **R**SPO1 CM, **N**oggin, **N**icotinamide, **E**GF, **A**83-01, **H**eregulinβ-1, **Y**-27632, **F**orskolin, **H**ydrocortisone, β-**E**stradiol	–	[69]
Fallopian Tube	**W**nt3A CM, **R**SPO1 CM, **N**oggin, **N**icotinamide, **E**GF, **F**GF10, **Y**-27632, **S**B431542	β-estradiol, progesterone	[71]
Endometrial	**R**SPO1, **N**oggin, **N**icotinamide, **E**GF, **F**GF10, **H**GF, **A**83-01	β-estradiol, progesterone, cAMP, PRL, hPL, HCG	[78]
	**W**nt3A CM, **R**SPO1 CM, **N**oggin, **N**icotinamide, **E**GF, **F**GF10, **A**83-01, **Y**-27632, **S**B202190, **I**TS, β-**E**stradiol	β-estradiol, progesterone	[77]
	**R**SPO1, **N**oggin, **E**GF, **A**83-01, **C**HIR99021, **P**GE2	β-estradiol	[79]
Cervical	Endocervix: **W**nt3A CM, **R**SPO1 CM, **N**oggin, **N**icotinamide, **E**GF, **F**GF10, **S**B431542, **Y**-27632	–	[94]
	Ectocervix: **N**oggin, **N**icotinamide, **E**GF, **F**GF10, **S**B431542, **Y**-27632, **F**orskolin, **H**ydrocortisone		
Trophoblast	**R**SPO1, **N**oggin, **E**GF, **H**GF, **A**83-01, **C**HIR99021, **P**GE2	Withdrawal of RSPO1 and CHIR99021	[100]
	**R**SPO1, **N**oggin, **E**GF, **H**GF, **F**GF2, **A**83-01, **C**HIR99021, **Y**-27632, **P**GE2	Adapted from Okae 2018: 2-mercaptoethanol, BSA, ITS-X supplement, NRG1, A83-01, KOSR; after cell outgrowth (d7-10) NRG1 was omitted	[101]

The medium composition for culturing organoid models of normal FRT tissue and placenta are listed. The growth factors and inhibitors are shown and the basal medium components (e.g., DMEM/F12, N2, B27, N-acetyl cysteine, Hepes, antibiotics) are not listed.

*RSPO1* R-spondin 1, *EGF* epidermal growth factor, *FGF* fibroblast growth factor, *HGF* hepatocyte growth factor, *ITS* insulin transferrin selenium, *A83-01* TGFβ receptor inhibitor, *SB431542* TGFβ receptor inhibitor, *SB202190* p38 MAPK inhibitor, *Y-27632* ROCK signaling inhibitor, *CHIR99021* GSK-3 inhibitor, *PGE2* prostaglandin E2, *cAMP* 8-Bromoadenosine 3’5’-cyclic monophosphate, *PRL* Prolactin, *hPL* human Placental Lactogen, *HCG* Human Chorionic Gonadotropin, *NRG1* Neuregulin 1, *KOSR* Knockout Serum Replacement, *BSA* Bovine Serum Albumin, *CM* conditioned medium.

^a^Excluding basal medium components.

### Fallopian tube

Fallopian tubes are bilateral ducts that connect the uterine body to the ovaries. In humans, fallopian tubes consist of four segments: the fimbriated ends bordering the ovaries (infundibulum), the ampulla, the ampulla–isthmus junction, and the isthmus (Fig. 2). There are characteristic folds in the epithelium along the entire length of the fallopian tubes. The fallopian tubal epithelium contains secretory (PAX8^+^, OVGP1^+^) and ciliated columnar (FOXJ1^+^, TUBB4A^+^) cells. The highest number of ciliated cells is in the fimbriae (up to 80%), decreasing to 30% in the isthmus [70]. These cilia capture the oocyte when it is released at ovulation and guide it down into the ampullary–isthmus junction for fertilization by sperm and then continue to guide the zygote into the uterus. The secretions from the secretory cells contribute by enhancing oocyte/zygote motility [70].

The first tissue-derived organoids of the FRT were from human fallopian tubes [71]. Cell isolates were prepared by enzymatic digestion and these were grown as monolayers until 70% confluence and then embedded into Matrigel (Table 2). The organoids recapitulate the in vivo phenotype functionally and phenotypically as demonstrated by their ability to respond to hormones (estrogen and progesterone), and by the presence of cilia, secretions, and folding of epithelium. They can be stably expanded long-term and are bipotent, giving rise to both secretory (PAX8^+^) and ciliated (TUBB4A^+^) cells. Active WNT and NOTCH signaling are required for the maintenance of the stem cells (Table 3). On the other hand, inhibition of NOTCH signaling leads to a decrease in Ki67^+^ proliferative cells with an increase in ciliated cells and upregulation of genes involved in ciliogenesis, *ARMC4, DNAI1, FOXJ1*, and *LRRC6* [71].

### Endometrium

**Fig. 3:**
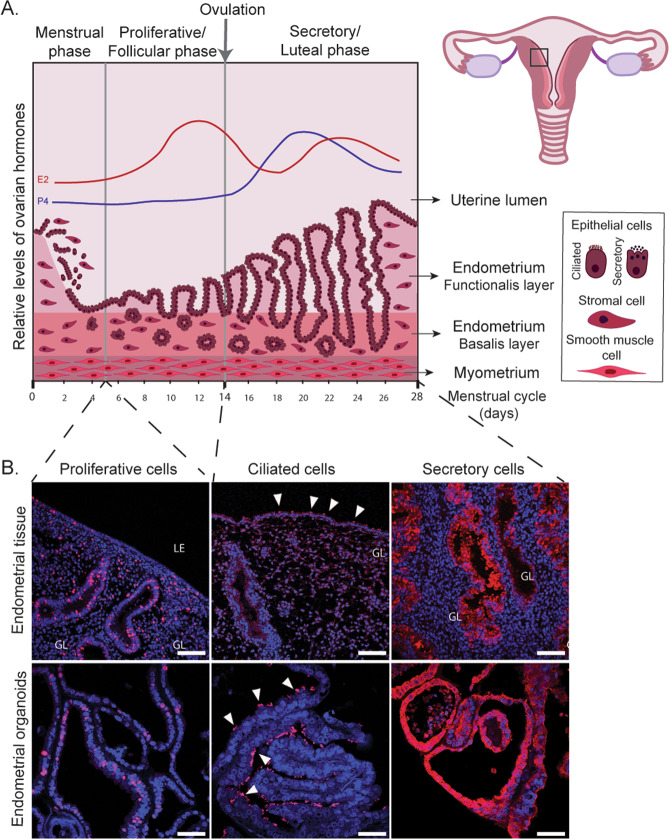
Endometrial organoids recapitulate essential features of proliferation and differentiation of human endometrium. **a** The endometrium is organized into two layers, the functional layer adjacent to the uterine lumen and the basal layer adjacent to the myometrium. It undergoes cyclical growth, differentiation, and shedding under the influence of ovarian hormones, estrogen (E2) (red line), and progesterone (P4) (blue line). The menstrual phase is followed by an E2 dominated proliferative phase. Ovulation marks the start of the secretory phase during the decidualization of the endometrium. In the absence of implantation hormone levels drop, resulting in shedding of the functional layer. The different cell types are depicted in the box. **b** Immunofluorescence of endometrial tissue (in vivo) and organoids (in vitro) to visualize proliferative (Ki67 positive, red), ciliated (acetylated-a-tubulin positive, red), and secretory cells (PAEP positive, red). Nuclei are counterstained with Dapi (blue). White arrowheads indicate ciliated cells. Scale bars, 100 μm (in vivo), 50 μm (in vitro). LE luminal epithelium, GL glands.

The endometrium, the mucosal lining of the uterus, is essential for reproduction in mammals and defects in its function are associated with implantation failure and pregnancy disorders [72, 73]. In humans, it is organized into the functional layer facing the uterine cavity and the underlying basal layer, which is adjacent to the myometrium (Fig. 3a). The functional layer is covered by a luminal epithelium from which the glandular epithelium invaginates into the stroma reaching as far as the basal layer. Whilst the luminal epithelium provides the site of implantation for the embryo, the glandular secretions support the development of the conceptus during the early weeks of pregnancy [74, 75].

The endometrium is highly dynamic and its regeneration, growth, and differentiation are exquisitely controlled by cyclical changes of ovarian hormones (Fig. 3a). The beginning of the cycle is marked by menstruation, during which the entire functional layer is shed. This is followed by the proliferative phase of the cycle dominated by estrogen from the ovarian follicle that stimulates the regeneration and growth of the functional layer. A peak of luteinizing hormone from the pituitary gland stimulates ovulation and progesterone production by the corpus luteum during the secretory phase. This drives a complex cascade of events leading to decidualization, the essential process to prepare the endometrium for pregnancy. In the absence of implantation, progesterone levels drop, resulting in menstruation, to start the cycle again [76].

Initial approaches to generate a 3D model of the endometrium consisted of isolating primary cells, growing them as a monolayer and transferring them into Matrigel to obtain spheroidal structures with columnar epithelium [42]. There have been several other reports but the expandability and molecular and functional characterization of the spheroids have not been investigated in detail [44–46]. Human and murine organoids of endometrial epithelial cells [77, 78] have now been generated and can be cultured long-term. These organoids are obtained by enzymatic digestion of endometrial tissues to release glandular fragments that are then embedded into Matrigel droplets (Table 2). Endometrial organoids are propagated in a medium that contains activators of WNT signaling (RSPO1, WNT3A, and CHIR99021) and growth factors (EGF, HGF, and FGF10) to promote proliferation. Inhibitors of TGFβ and BMP signaling pathways (A83-01 and Noggin, respectively) are added to prevent differentiation, as well as nicotinamide for the establishment of organoids [77–79] (Table 3). The addition of an inhibitor of p38 MAPK together with low levels of 17β-estradiol has also been reported [77]. Endometrial organoids are spheroidal with a central lumen surrounded by a single epithelial layer. They have cellular heterogeneity, self-organize, are chromosomally stable, have clonogenic capacity and similar molecular signatures to the glands in vivo as shown by the expression of glandular markers (MUC1, ECAD, KRT7, EPCAM, FOXA2, and Pan-KRT) [77, 78]. Endometrial organoids can be robustly established with high success rates from proliferative, secretory, menopausal, and pregnant endometrium (decidua) [78].

Endometrial organoids recapitulate the morphology and functions of the human endometrium in vivo by responding to estrogen and progesterone (Fig. 3b). Treatment with estrogen increases the number of Ki67^+^ cells, a marker of the proliferative phase [77] and stimulates the generation of ciliated cells [79]. Combined estrogen and progesterone treatment results in nuclear expression of their receptors, ESR1 and PGR, and production of characteristic secretory phase proteins, progestagen-associated endometrial protein (PAEP) and secreted phosphoprotein 1 (SPP1). Additional treatment with signals from decidualized stroma (prolactin and cyclic AMP) and the placenta (chorionic gonadotropin and placental lactogen) further stimulates them to acquire a phenotype typical of decidual glands [78].

The clonogenic ability of endometrial organoids provides an opportunity to explore putative markers of human endometrial stem cells [80]. CDH2, a component of the WNT signaling pathway, is proposed as a marker of the sparse endometrial progenitor cells thought to be located in glands in the basal layer, and CDH2^+^ cells demonstrated a higher clonogenic and proliferative capacity [81]. Single-cell RNA sequencing of endometrial organoids revealed proliferating, secretory, ciliated, and putative stem cell populations [82]. The bipotential ability of the organoids also allows the study of the epithelial differentiation process. Similarly to the organoids from the fallopian tube, inhibition of NOTCH signaling is involved in ciliogenesis that is dependent on the presence of estrogen [79]. As an example of how the organoids can be used to study endometrial physiology, the role of the mechanosensitive ion channel PIEZO1 was investigated and shown to be involved in the establishment of a dialogue at the maternal–fetal interface that could act as a potential target for promoting successful implantation [83].

### Cervix

**Fig. 4:**
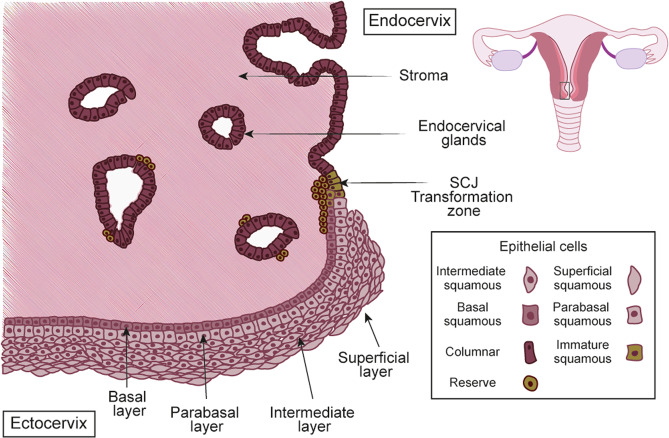
The anatomy of the human cervix. The cervix consists of two distinct epithelia; the columnar epithelium of the endocervix and stratified epithelium of the ectocervix, which merge in the squamocolumnar junction (SCJ). The reserve cells (in green) are localized under the columnar epithelium and are believed to regulate the process of metaplasia, which results in the formation of a new stratified epithelium, producing the transformation zone (TZ).

The cervix connects the uterus to the upper vagina. The increase in estrogen at ovulation causes softening and production of cervical fluid allowing smooth transit of sperm [84]. The cervix is divided into three regions: the ectocervix, squamocolumnar junction (SCJ), and endocervix (Fig. 4). The ectocervix is the outer part of the cervix that protrudes into the vagina and has a stratified non-keratinizing squamous epithelium composed of basal, parabasal, intermediate, and superficial layers. These layers can be distinguished by expression of specific keratins: superficial and intermediate layers are KRT4^+^ and KRT13^+^; parabasal layers are KRT13^+^, KRT14^+^, and occasionally KRT10^+^; and basal layer express KRT5, 14, 18, and 19 [85, 86]. The endocervix, which is continuous with the endometrium, has a monolayer of mucin-producing, columnar epithelial cells that express KRT7, 8, and 18, interspersed with sporadic ciliated cells [87, 88].

The SCJ is a unique site because it is the junction between two different epithelial types. It is formed ~20–24 week fetal stage in response to maternal hormones [89], and undergoes continuous remodeling during puberty, pregnancy, and menopause by a process of squamous metaplasia [90], creating an area of transition between the two epithelial lineages called the transformation zone (TZ) (Fig. 4). Metaplastic change starts with the active division of subcolumnar KRT17^+^ (reserve) cells located in the endocervix. Subsequently, the reserve cells differentiate into layers of immature, stratified epithelium, where both squamous and columnar components can be detected; these progressively mature to blend with the original ectocervix, making up the TZ [91–93].

The term ‘cervical organoids’ describes both endo- and ecto-cervical organoids. Organoids have been generated and cultured long-term from normal adult cervical tissues that recapitulate the site-specific epithelial architecture of the cervix [94] (Table 2). Differentiation towards the endocervical lineage requires WNT signaling, whilst its inhibition is critical for stratification [94] (Table 3). Furthermore, NOTCH signaling was key for squamous lineage differentiation at the SCJ. The endocervical organoids express KRT7, whilst the ectocervical ones are positive for basal markers KRT5 and TP63. Organoids have also been derived from the cervical SCJ and grown in a medium containing EGF, RSPO1, Noggin, Y-27632, and Jagged-1 [95]. MMP7 and AGR2 are expressed in these organoids, which are also KRT17^+^. Dual cell populations are present; endocervix/columnar cells, which are PAS^+^, P40^−^ and an ectocervix/squamous cells which are PAS^−^, P40^+^. The percentage of each type was variable. Further work must be carried out to generate a more robust SCJ organoid model.

### Placenta

**Fig. 5:**
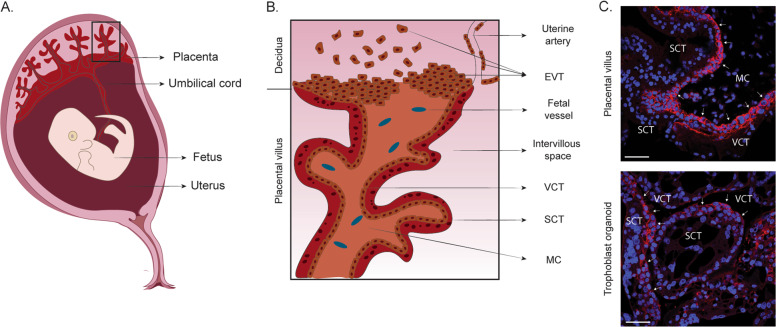
The pregnant uterus of the first trimester and the placenta. **a** The placenta is firmly embedded into the pregnant lining of the uterus, the decidua, on one side and on the other, is connected to the fetus via the umbilical cord. **b** The placenta is made up of a system of branching villi that is covered by a bilayered epithelium—a multinucleated outer layer called the syncytium (SCT) and the mononuclear epithelium, the villous cytotrophoblast (VCT). The mesenchymal core (MC) of the villi contains stromal and endothelial cells and macrophages. At the tips of the villi, extravillous trophoblast (EVT) attach and invade into the stroma and the spiral arteries of the decidua. **c** Trophoblast organoids derived from human first-trimester placenta. Immunofluorescence of a placental villus (in vivo) and trophoblast organoids (in vitro) for VCT marker EPCAM (in red). VCT is shown with white arrows. Nuclei are stained with Dapi (blue). Scale bars, 50 μm.

The placenta is an extra-embryonic organ responsible for nourishing and protecting the developing conceptus during its life in utero. Its development begins when the blastocyst (preimplantation embryo) segregates into the inner cell mass and the trophectoderm. The trophectoderm comes into direct contact with the luminal epithelium of the endometrium at implantation and gives rise to all the trophoblast lineages of the placenta: an inner villous cytotrophoblast (VCT) and an outer syncytiotrophoblast (SCT) responsible for maternal–fetal exchange and production of pregnancy hormones and the extravillous trophoblast (EVT) which migrate out of the villi and attach and invade into the decidua, the pregnant endometrium [96] (Fig. 5a). In humans, the placenta is intimately associated with the decidua. The EVT transforms the spiral arteries from outside via the stroma (interstitial invasion) and inside (endovascular invasion). This arterial transformation is essential to supply all the nutrients and blood to the developing fetus. It is fundamental for the success of the pregnancy as the major pregnancy disorders show defects in this process. The development of the placenta is directed by signals from the decidua including glandular secretions, which provide the initial source of nutrition before the onset of maternal–fetal circulation into the intervillous space [97].

Although animal models and cell lines have been essential tools in placental research, they have several limitations. The deep invasion of trophoblast in human placentation is only seen in the great apes and is not fully modeled in mice [98]. Primary human trophoblast cells cannot be cultured long term and choriocarcinoma cell lines have an abnormal karyotype [96]. Human trophoblast stem cell (hTSC) lines were recently generated from blastocysts and first trimester placentas [99]. hTSC are maintained by activated WNT and MAPK signaling (via EGF) and inhibition of TGFβ, ROCK and histone deacetylase. These cells have clonal ability, differentiate into the two main trophoblast lineages, SCT and EVT, and satisfy the key criteria of human trophoblast in vivo, providing definitive evidence that they are indeed trophoblast. However, hTSC grow as a monolayer and lack the architectural organization of the placenta. To model the placental villi, trophoblast organoids were established from first-trimester placentas (Table 2) [100, 101]. Under conditions that promote WNT signaling, trophoblast organoids grow with an inner syncytial mass and an outer proliferative VCT (Fig. 5b). Switching the medium, by either omitting WNT activators or using differentiation medium used for 2D hTSC [99], trophoblast organoids can be stimulated to generate EVT (Table 3). They are genetically stable long-term and their transcriptomes and epigenetic signatures closely resemble first-trimester trophoblast in vivo. Importantly, trophoblast organoids showed key functions of the placenta: their secretome was investigated by mass spectrometry and many placental products are identified and the EVT are highly invasive and burrow their way through Matrigel [101]. These organoids provide an experimental model to investigate placental development and the cell lineage relationships of the different trophoblast subpopulations. They also provide the opportunity to study the cellular and signaling interactions between the placenta and the decidua.

## Organoids for the study of disorders of the FRT

### Endometriosis and endometrial hyperplasia

Organoids can be established from pathological samples and provide powerful tools to study diseases ex vivo although the culture conditions often need optimizing [69, 78, 95, 102–111] (Table 4). A common benign condition is endometriosis where ectopic foci of the endometrium containing both glands and stroma are found in the peritoneal cavity, ovary, and cervix. Active WNT and EGF, with inhibition of BMP signaling, are necessary for efficient growth of organoids from these lesions. Organoids from ectopic sites of endometriosis express genes linked to stemness (*SOX9, LGR6*) and metalloproteinase function (*MMP2*), and show luminal invasion, a common characteristic of endometriosis. Somatic mutations in genes associated with endometrial carcinoma (*CTCF, EP300, ZNF471*) were found in organoids from late-stage endometriotic lesions. When engrafted into mice, they give rise to ESR1^+^ and PR^+^ lesions. Using the same medium composition for endometriosis, organoids can also be established from endometrial hyperplasia. This is a condition usually found in anovulatory women exposed to estrogen without progesterone and is characterised by an abnormally thick endometrium. Organoids from endometrial hyperplasia  display high proliferative capacity and similar molecular characteristics as the primary tissue, including absence of TP53 expression. Mutations in mismatch DNA repair genes (*MSH2, MSH6*) were observed in organoids derived from women with Lynch syndrome [108].

**Table 4:** Organoids derived from pathological tissues of the human FRT.

Tissue	Pathology	Applications	Medium components^a^	Reference
Ovaries	Ovarian carcinoma	Drug screening (multidrug assay)	b**F**GF, **E**GF, **I**GF-I, β-**m**ercaptoethanol, **H**eparin, **H**ydrocortisone, **I**nsulin **Y**-27632, β-**E**stradiol	[102]
		Prediction of DNA repair in response to inhibitors	**R**SPO1, **No**ggin, **N**icotinamide, **E**GF, **F**GF-10, **F**GF2, **P**rostaglandin E2, **A**83–01, **S**B202190	[103]
		Characterization, Gene editing, Drug screening, In vivo drug sensitivity	**R**SPO1, **W**NT CM^d^, **N**oggin, **N**icotinamide, **E**GF, **F**GF-10, **A**83-01, **H**eregulin-β, **Y**-27632, **F**orskolin, **H**ydrocortisone, β-**E**stradiol	[69]
		Characterization, Drug sensitivity	**R**SPO1, **N**oggin, **E**GF, **Y-**27632, **J**agged-1	[104]
		Multi-drug screening	**P**rEGMTM or **M**ammoCult^e^	[105]
		Characterization, Optimization of culture conditions	**N**icotinamide, **E**GF, **S**B431542, **Y**-27632, **B**MP2	[106]
Fallopian tube	Chlamydia infection	Characterization, Investigation of chronic infection	**R**SPO1, **W**nt3A, **N**oggin, **N**icotinamide, **E**GF, **F**GF-10, **S**B431542, **Y**-27632	[107]
Endometrium	Endometriosis	Characterization, Analysis of ion channels	**R**SPO1, **N**oggin, **N**icotinamide, **E**GF, b**F**GF, **F**GF-10, **A**83-01, **S**B202190 (low), **I**TS, β-**E**stradiol^b^, Y-27632^c^	[108]
	Endometrial hyperplasia			
	Lynch syndrome			
	Endometrial carcinoma	Evaluation of apoptotic effects of verteporfin	**E**GF, b**F**GF, **I**nsulin, **B**SA	[109]
		Evaluation of growth inhibitory effects of drugs	**N**icotinamide, **A**83-01, **S**B202190, **Y**-27632, β-**E**stradiol	[110]
		Characterization	**R**SPO1, **N**oggin, **N**icotinamide, **E**GF, **F**GF10, **H**GF, **A**83-01	[78]
		High-throuput multidrug screening	**F**BIM002 medium	[111]
		Characterization	**R**SPO1, **N**oggin, **E**GF, **Y**-27632, Jagged-1	[104]
		Characterization, Drug screening, Analysis of ion channels	**R**SPO1 (low), **N**oggin, **N**icotinamide (high), **E**GF, **I**GF-1, **H**GF, **A**83-01, **S**B202190, β **E**stradiol, Y-27632^c^	[108]
Cervix	Cervical clear cell carcinoma	Characterization, Drug screening	**R**SPO1, **N**oggin, **E**GF, **Y**-27632, Jagged-1	[115]

Organoid models of pathologies of the human FRT are shown together with their applications. The medium composition with growth factors and inhibitors are shown and the basal medium components (e.g., DMEM/F12, N2, B27, N-acetyl cysteine, Hepes, and antibiotics) are not listed.

*RSPO1* R-spondin 1, *EGF* Epidermal Growth Factor, *FGF* fibroblast growth factor, *HGF* hepatocyte growth factor, *IGF-1* Insulin-like Growth Factor-1, *ITS* Insulin Transferrin Selenium, *A83-01* TGFβ receptor inhibitor, *SB431542* TGFβ signaling inhibitor, *SB202190* p38 MAPK inhibitor, *Y-27632* ROCK signaling Inhibitor, *BSA* bovine serum albumin.

^a^Excluding basal medium components.

^b^Only for organoid expansion.

^c^Only for organoid formation or dissociation at passaging.

^d^WNT-conditioned medium was used only for some tumor organoid lines.

^e^Serum-free commercial media (PrEGMTM Prostate Epithelial Cell Growth Medium, MammoCult; for culture of mammospheres from normal human primary breast tissues and tumorspheres from human breast cancer cell lines.

### Carcinomas of the FRT

Several organoid models have now been derived from carcinomas arising in the FRT (Table 4). Ovarian carcinomas are heterogeneous with high grade serous ovarian carcinoma (HGSOC) being the deadliest and most common type [112]. It seems that HGSOC originates from the epithelium of the fimbriae of the fallopian tube [113], although others have proposed the source is the OSE [114]. 3D cultures from solid tumors, ascitic, and pleural fluid of patients with ovarian carcinoma were generated [103]. Whilst these cultures displayed clonogenic capacity and morphological and molecular similarity to the primary tumors, they could only be expanded short-term. In another study, a biobank of organoids from pre-malignant and malignant ovarian neoplasms was established [69]. These organoids recapitulate morphological (nuclear and cellular atypia), phenotypic (PAX8^+^, TP53^+^), and genomic features (mutations in KRAS, BRAF, cell cycle genes, and TP53), capture tumor heterogeneity and can be expanded long-term. However, these culture conditions are still suboptimal and HGSOC organoids grow slowly. Growth and long-term expansion has now been achieved by using media with low WNT and active BMP signaling to maintain stem cells in the cultures (Table 4) [106]. These conditions are also required for the stable growth of another model of HGSOC that were derived by the stable triple knockdown of *TP53*, *PTEN*, and *RB* in fallopian tube organoids [106].

Similarly, organoids can be derived from different stages of endometrial neoplasms that capture the phenotypic and genetic heterogeneity (Table 4). Low WNT but high p38 MAPK signaling, both associated with cell proliferation and differentiation, together with high concentration of β-estradiol, addition of growth factors (IGF1, HGF) and lipids enhance the expandability of organoids derived from endometrial carcinomas [108]. Organoids from low-grade endometrial carcinomas bear mutations in tumor suppressor genes (*PTEN, CTCF,* and *ARID1A*) and the β-catenin coding gene (*CTNNB1*), resulting in continuous activation of the WNT pathway. In contrast, organoids from high-grade endometrial carcinomas were characterized by downregulation of glandular markers (*ESR1, FOXA2*) but upregulation of EMT-associated genes (*CXCR4, TWIST1, ZEB1,* and *CDH2*) [108]. These organoids can be orthotopically engrafted into murine uterine horns where the histological and molecular features of the original lesion are retained with the potential to metastasize [108].

Organoids derived from clear cell carcinoma, a rare tumor of the cervix could be propagated for more than 6 months, retained 2 out of 3 mutations (*MLH1* and *TFE3)* detected in the original tumor and typical markers, HNF1‐β, TP53, and Ki‐67 [115] (Table 4). Their appearance was similar to the original tumor with atypical cells with clear cytoplasm. No organoid models of squamous cervical carcinoma have been reported.

### Drug screening on patient-derived organoids

Patient-derived organoids from pathologies of the FRT can be frozen and thawed allowing generation of extensive biobanks, which can be used for drug sensitivity screening and ultimately for personalized medicine [69, 102, 104, 105, 108]. When treated with platinum/taxane, drugs commonly used for treating ovarian cancer, different organoid lines showed differential drug responses. Sensitive and resistant organoids correlated with the grade of the tumor (high or low) and the degree of chemoresistance previously noted in the patients [69]. Drug responses have also been examined in vivo in mice xenografted with organoids of ovarian carcinomas [69]. When the mice were treated with gemcitabine, a nucleoside analog commonly used for the treatment of HGSOC, proliferation, and invasion of the tumors was restricted.

Similarly, patient-specific responses were also observed in organoids derived from endometrial carcinoma treated with standard chemotherapeutic compounds [108, 110, 111]. The resistance of the organoids to cisplatin and paclitaxel echoed the patients’ clinical response to treatment [111]. Another report described the induction of apoptosis in organoids from endometrial carcinoma in response to verteporfin, a drug which inhibits the HIPPO pathway [109]. Similarly, napabucasin, an inhibitor of STAT3 signaling hampered their growth [110]. This variability in drug response may be due to the lack of stromal or immune cells in these organoid models [116, 117], which could be resolved with the development of co-culture systems.

## Applications and future perspectives of FRT organoids

### Genome engineering

**Fig. 6:**
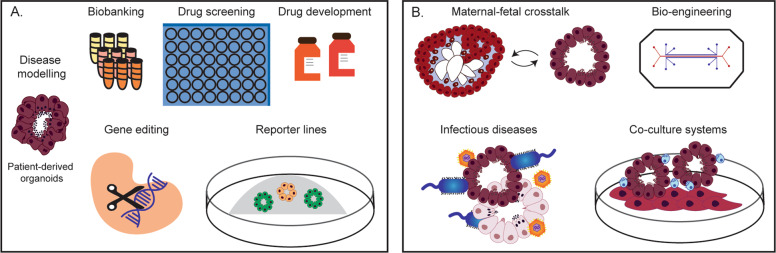
Applications of tissue-derived organoids of the female reproductive tract and placenta. **a** Organoid systems can be used for studying the physiology and pathologies of the FRT. They can be used as. tools for testing drug responses and drug development. Gene function can be assesed by CRISPR/Cas9 based genetic engineering of organoids. **b** The possibility to combine organoids with different cell types as well as pathogens will allow studies on their interactions. Bioengineering methods may allow the generation of more complex tissue-like models that include non-epithelial populations such as fibroblasts, immune and endothelial cells. The interactions between two different tissues can also be studied by co-culture of organoids, which is of particular relevance for maternal–fetal crosstalk using placental (trophoblast) and endometrial organoids.

Combining techniques to engineer genomes of organoids from the FRT open up new possibilities to study its physiology and disease (Fig. 6a). There is still much to learn about epithelial regeneration, maintenance, and differentiation of the FRT and it is evident that CRISPR/Cas9 gene editing technology will help address many outstanding questions. It allows site-specific targeting to disrupt or modify a genetic locus of interest. For example, transcription factors involved in proliferation and differentiation of the epithelial cells of the FRT can be targeted to study their functions. It can be used for creating reporter lines and performing lineage tracing experiments to identify progenitor populations of the different regions of the FRT. In a forward genetics approach, specific mutations can be introduced to study the etiology of endometrial and cervical carcinomas as previously done for targeting genes involved in ovarian cancer using fallopian tube organoids [69].

### Assisted reproductive techniques

One of the exciting prospects of the generation of organoids from the different regions of the FRT is the possibility of personalized medicine (Fig. 6a). This is especially relevant for studying infertility. Even though all patients undergoing in vitro fertilization (IVF) have the same hormonal treatment, the cause of the infertility is unexplained in ~30% of cases [118]. Organoids could be generated from endometrial biopsies from women undergoing IVF and exposed to these hormonal regimes to investigate whether differential responses may be one of the possible causes. Treatment regimens could then be tailored to each patient. A similar approach could be used for treating endometriosis where the eutopic endometrium is often progesterone resistant and patients have fertility issues [119].

Further optimization of organoid models of the healthy ovarian epithelium could prove useful in treating suboptimal infertility caused by anovulation that occurs in women with polycystic ovary syndrome. Ovulation induction is one of the most common therapeutic approaches. This comes with risks of ovarian hyperstimulation syndrome [120], causing patients a range of symptoms (abdominal pain, vomiting, fluid accumulation in the abdomen, and lungs) that may require hospitalization. In vitro maturation of eggs, using ovarian organoids would be a less invasive and risky approach for assisted reproductive technology.

### Co-culture models of FRT

Tissue-derived organoids of the FRT include only the epithelial compartment and thus do not fully  reflect the cellular complexity of the native tissues. For the study of cellular interactions, multicellular organoid co-culture systems are necessary (Fig. 6b). The interdependence of endometrial epithelial cells with stromal cells in the endometrium has been studied extensively using animal models but this is difficult to model in humans [121, 122]. Stromal cells respond to estrogen and progesterone by producing prolactin and IGFBP1, which act on the glands to stimulate secretions [123]. In an organotypic approach, glandular epithelial cells embedded in Matrigel were cultured on a monolayer of stromal cells to investigate the effects of steroid hormones and anticancer drugs on the epithelial compartment [124]. Alternative methods for epithelial-stromal co-cultures utilize scaffolds. This also cirumvents the use of Matrigel which is not chemically defined and has batch-to-batch variation [125]. Recently, a porous collagen-based co-culture model of the epithelial and stromal cells of the endometrium was developed with both cell types being functionally responsive to hormones [126]. In future, the architecture of these scaffolds could be manipulated to allow the formation of gland-like structures that closely resemble the native tissue. Careful comparison with information about the in vivo environment is essential to prove the validity of the models [127], and to identify the characteristics of the niche that target epithelial populations [128]. Although these systems show promise in understanding the endometrium as a whole, there are still issues to resolve like finding media appropriate for all cell types.

Immune cells play a major role in the homeostasis and function of the FRT. In the endometrium, progesterone upregulates IL-15 that is trans-presented by stromal cells to stimulate the proliferation and differentiation of uterine natural killer cells [129]. How these hormonally-regulated, distinctive, uterine lymphocytes play a role in epithelial regulation and function has been challenging to study. Co-culture methods that combine epithelial organoids with immune cells provide insights into normal intestinal homeostasis and diseases including gastric, pancreatic, colorectal, lung, and breast neoplasms [130]. Similar co-culture systems of organoids of FRT with immune cells will clearly be an important advance (Fig. 6b). They will also be important in understanding behavior of tumors of the FRT where the microenvironment plays a crucial role in the development and progression of the disease [131].

A further use of multicellular culture models of the FRT would be to explore early developmental events in humans that are impossible to study in vivo (Fig. 6b). Though such studies need to be scrutinized appropriately and ethical considerations must be taken into account [132]. An artificial endometrium could be used to model implantation in a dish [133, 134]. Previous attempts to study the attachment of the blastocyst or trophoblast onto the endometrium [45, 46, 48, 135] used epithelial monolayers superimposed on stromal cells, thus not recapitulating the in vivo 3D glandular structure. An important application of trophoblast organoids that differentiate to invading EVT, will be to study molecular and functional interactions with decidual cells (uterine NK, macrophages, epithelial, and stromal cells) in vitro.

### FRT organoids and infection

Organoids are a practical model to study infectious diseases [65]. Tubal infections caused by sexually transmitted diseases, misuse of intrauterine contraceptive devices, or post-miscarriage, can lead to tubal fibrosis, loss of patency, and infertility [136]. Such infections have also been linked to cancer [137]. The most common pathogen is *Chlamydia trachomatis*. A microarray analysis of infected organoids during the acute phase of a Chlamydia Ctr serovar D infection shows prolonged activation of leukemia inhibitory factor signaling that could play a role in maintaining stem cell identity (Table 4) [107]. The robust activation of paracrine networks controlling not only cell growth and proliferation, but also differentiation and cell fate, suggests that Ctr infection has pervasive long-term consequences on the epithelium.

Pathogens of the lower reproductive tract including vaginal bacterial species (lactobacilli and vaginosis associated bacteria) can ascend to the endometrial cavity [138]. Infection with *Neisseria gonorrhoeae* of 3D epithelial cultures derived from endometrial adenocarcinoma resulted in upregulation of proinflammatory mediators and morphological changes to the host cells [139]. Endometrial organoids will be a valuable model to investigate chronic endometritis commonly caused by *Chlamydia trachomatis*, *Neisseria gonorrhoeae,* and *Trichomonas vaginalis* [140]. Co-culture of immune cells with the organoids will increase understanding of the pathogenesis of endometritis (Fig. 6b).

The major disease affecting the cervix is dysplasia and subsequent carcinoma caused by high-risk human papilloma virus (HR-HPV) [90]. Modeling the normal metaplastic change from glandular to squamous epithelium and the response of the epithelium to HPV infection with progression to carcinoma will provide information about the dynamics of epithelial renewal. In addition, cervical organoids can offer a system to study sexually transmitted diseases (*Chlamydia trachomatis, Neisseria gonorrhoeae, Trichomonas vaginalis,* and herpes simplex virus).

## Conclusions

Throughout adult reproductive life, the FRT undergoes constant remodeling under the influence of pituitary and ovarian hormones and, if pregnancy occurs, it goes through dramatic changes driven by placental hormones. Disruption of all these complex, exquisitely controlled processes results in a diverse range of pathologies that together affect a large number of women worldwide. Although much is known about hormonal changes occurring in the FRT, it is mostly descriptive without detailed molecular and cellular information. Now available are essential experimental organoid models of the FRT that recapitulate the original tissues (healthy or pathological). Although organoid cultures are more labor intensive and costly compared with standard 2D culture, they can be set-up with relative ease allowing wide-spread use. There are still many questions to be answered regarding the FRT organoids. Efforts are now being made to define the common issues affecting all organoids systems such as reproducibility, standardization, and diligence validation [141]. This is an exciting time for reproductive research as recent progress paves the way for opportunities to improve women’s wellbeing and reproductive health.
